# A New Presentation: Aphallia, Vesicoureteral Reflux, Rectovesical Fistula, and Adrenal Insufficiency

**DOI:** 10.1155/2020/8826520

**Published:** 2020-11-23

**Authors:** R. El Qadiry, A. Lalaoui, H. Nassih, A. Bourrahouat, I. Ait Sab

**Affiliations:** Pediatric B Department–Mother-Child Pole, Mohammed VI University Hospital, Marrakesh, Morocco

## Abstract

Aphallia or penile agenesis is a rare congenital malformation with an estimated incidence rate of 1 in 10 to 30 million births. More than half of aphallia cases have associated anomalies including caudal axis, cardiovascular, genitourinary, and gastrointestinal anomalies. The penile agenesis associated with adrenal insufficiency has never been reported in an infant. We report a rare case of a newborn that was diagnosed as a case of aphallia with vesicorectal fistula and vesicoureteral reflux, complicated by adrenal insufficiency with salt-wasting crisis.

## 1. Introduction

Aphallia is an extremely rare congenital anomaly; it has an estimated incidence rate of 1 in 10 to 30 million births [1]. It is believed to result from the absence or a failure in the development of the genital tubercle.

Aphallia may occur alone or it may have associated with other congenital anomalies. Various associated congenital anomalies have been reported in the literature. More than half of these patients have associated anomalies including genitourinary (54%) and gastrointestinal tract anomalies and developmental defects of the caudal axis [2].

Our case had a unique presentation because there is no report of the penile agenesis associated with bilateral vesicoureteral reflux, vesicorectal fistula, and adrenal insufficiency in an infant.

## 2. Case

A term male infant with a birth weight of 3 kg 400 g was referred to our department with the diagnosis of aphallia. The child was born of an outbred marriage with an uncomplicated prenatal period. There was no family history of birth defects or ingestion of teratogen.

**Figure 1:**
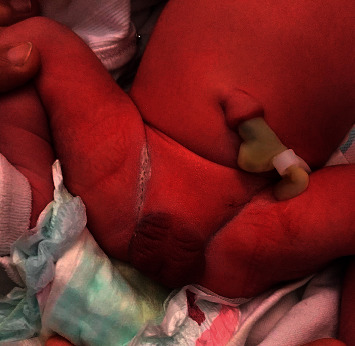
Photograph illustrating aphallia with a normal scrotum.

**Figure 2:**
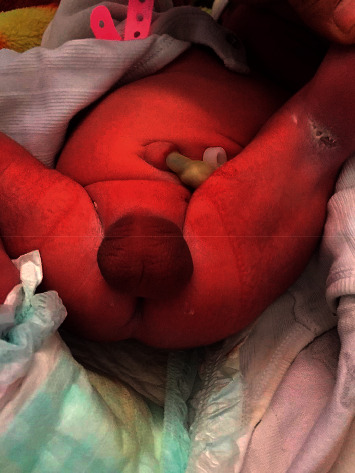
The anal opening is located normally.

The physical examination showed a well-developed male, except for complete absence of a phallus. The scrotum was normal, and the newborn had bilaterally descended testicles (Figure 1). The anal opening was located normally (Figure 2). The urethral opening was not visible anywhere in the perineum. The rest of general and systemic physical examination was normal.

**Figure 3:**
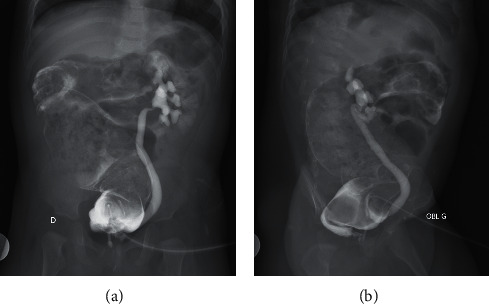
Grade 3 unilateral vesicoureteral reflux (VUR) with a thin tract opening in the rectum.

At the time of the investigation, the child had a 46, XY karyotype. Ultrasonography of the abdomen showed kidneys of normal size with regular contours and bilateral ureterohydronephrosis more prominent on the left side. The renal function tests were normal. The cystostomy operation was performed after the physical examination and ultrasound results on the same day. Cystourethrography revealed grade 3 unilateral vesicoureteral reflux (VUR) with a thin tract opening in the rectum, suggesting an urethrorectal fistula (Figure 3).

A month later, the infant presented to our hospital with a history of 15-day vomiting for multiple episodes that were nonbilious and non-blood-stained. Neither fever nor irritability was noted.

On general examination, he was lethargic with sunken eyes, dry oral mucosa, and depressed anterior fontanelle. Examinations of the respiratory, cardiovascular, and neurological system were normal.

Investigations showed a negative septic screening (blood cultures, cerebrospinal fluid, and CBEU) with deranged electrolytes. His serum Na+ was 113 mEq/l (N: 135–145), K+ was 6.7 mEq/l (N: 3.5–5.1), and HCO3 was 15 mEq/L (N: 21–29 mEq/L). Renal function tests and capillary blood glucose were normal.

A probable diagnosis of adrenal insufficiency with the salt-wasting crisis was made according to examination and investigation, and the treatment commenced.

Blood was drawn and sent for steroid hormone measurements. The diagnosis of primary adrenal insufficiency was made in view of an elevated serum adrenocorticotropic hormone concentration at 310 pg/ml (N: 7.20–63.30) associated with a low cortisol concentration at 1.30 *µ*g/dl (N: 3.70–19.40).

Plasma renin concentration dramatically increased at 1894 mUI/ml (N: 2.8–39.9 mUI/ml) contrasting with a low aldosterone concentration at 12 pg/l (N: 8–172 pg/ml), confirming the clinical suspicion of the salt-wasting crisis. Steroid supplementation with hydrocortisone and fludrocortisone resulted in rapid improvement of his clinical condition.

Congenital adrenal hyperplasia (CAH), especially due to 3-beta-hydroxysteroid dehydrogenase deficiency, was excluded: dehydroepiandrosterone (DHEA) at 332 ng/ml (N < 400 ng/ml), 17-hydroxypregnenolone at <1.1 nmol/l (N < 10.8), 17-hydroxyprogesterone at 3.3 nmol/l (N: <7.5), Δ4 androstenedione at 2.9 nmol/l, and testosterone at 6.8 nmol/l (N: <13.8 nmol/l). Computed tomography (CT) scan of the abdomen showed normal adrenal gland eliminating congenital adrenal hypoplasia and aplasia. Molecular analysis was not performed; therefore, the etiology of adrenal insufficiency has not yet been found for the lack of means.

He was sent home on oral hydrocortisone, fludrocortisone acetate, and 0.9% sodium chloride with therapeutic education of his parents. They also received an emergency card.

## 3. Discussion

Aphallia or penile agenesis is an extremely uncommon congenital anomaly with a reported incidence of 1/10 to 1/30 million births. It develops as a result of the failure of the development of the genital tubercle into the phallus. This leads to the total absence of all three components of the penile shaft, i.e., both corpora cavernosa and spongiosum [3].

Diagnosis of aphallia includes the absence of the phallus, male karyotype, and normally developed scrotum and normal and frequently undescended testicles [4]. It should be differentiated from concealed penis, epispadias, hypospadias, micropenis, rudimentary penis, intrauterine amputation of the penis, and disorders of sexual development [5].

Aphallia may occur alone or it may have associated with other congenital anomalies. Various associated congenital anomalies have been reported in the literature. Associated genitourinary anomalies are seen in up to 54% of the patients. Yurtçu reported a case of aphallia with bilateral vesicoureteral reflux and vesicorectal fistula in an infant in 2019 [6]. Our case is differentiated from the case reported by Müslim Yurtçu because of its unique presentation with adrenal insufficiency. To the best of our knowledge and following a review of the PubMed database, we report a never-before described associated anomaly with PA making this report a unique contribution to the world literature.

Treatment of aphallia presents many challenges, and it involves a multidisciplinary approach. Our patient remained a boy as per the preference of the parents as well as the prevailing socioeconomic conditions. Cystostomy with vesicorectal fistula ligation may be an alternative to the conventional procedure in this case to prevent urinary tract infection. The penile reconstruction is a better option due to psychosocial implications for this patient.

## 4. Conclusion

Aphallia is a rare malformation that usually coexists with other serious anomalies incompatible with normal life. Our case is a new example of an associated malformation in the newborn with penile agenesis. Thus, a complete evaluation of all systems, including the adrenal glands, is necessary in patients with aphallia to rule out all of these abnormalities.
